# Structural and Functional Imaging Studies in Chronic Cannabis Users: A Systematic Review of Adolescent and Adult Findings

**DOI:** 10.1371/journal.pone.0055821

**Published:** 2013-02-04

**Authors:** Albert Batalla, Sagnik Bhattacharyya, Murat Yücel, Paolo Fusar-Poli, Jose Alexandre Crippa, Santiago Nogué, Marta Torrens, Jesús Pujol, Magí Farré, Rocio Martin-Santos

**Affiliations:** 1 Psychiatry, Institute of Neurosciences, Hospital Clínic, IDIBAPS, CIBERSAM, Barcelona, Spain; 2 Department of Psychiatry and Clinical Psychobiology, University of Barcelona, Barcelona, Spain; 3 Melbourne Neuropsychiatry Centre, The University of Melbourne, Melbourne, Victoria, Australia; 4 Department of Psychosis Studies, King’s College London, Institute of Psychiatry, London, United Kingdom; 5 Neuroscience and Cognitive Behavior Department, University of Sao Paulo, Ribeirao Preto, Brazil; 6 National Science and Technology Institute for Translational Medicine (INCT-TM, CNPq), Ribeirao Preto, Brazil; 7 Clinical Toxicology Unit, Emergency Department, Hospital Clínic, IDIBAPS, University of Barcelona, Barcelona, Spain; 8 Neuroscience Program, Pharmacology Unit and Drug Addiction Unit, IMIM-INAD-Parc de Salut Mar, Autonomous University of Barcelona, Barcelona, Spain; 9 Red de Trastornos Adictivos (RETIC), IMIM-INAD-Parc de Salut Mar, Barcelona, Spain; 10 Institut d’Alta Tecnologia-PRBB, CRC Mar, Hospital del Mar, Barcelona, Spain; Peking University, China

## Abstract

**Background:**

The growing concern about cannabis use, the most commonly used illicit drug worldwide, has led to a significant increase in the number of human studies using neuroimaging techniques to determine the effect of cannabis on brain structure and function. We conducted a systematic review to assess the evidence of the impact of chronic cannabis use on brain structure and function in adults and adolescents.

**Methods:**

Papers published until August 2012 were included from EMBASE, Medline, PubMed and LILACS databases following a comprehensive search strategy and pre-determined set of criteria for article selection. Only neuroimaging studies involving chronic cannabis users with a matched control group were considered.

**Results:**

One hundred and forty-two studies were identified, of which 43 met the established criteria. Eight studies were in adolescent population. Neuroimaging studies provide evidence of morphological brain alterations in both population groups, particularly in the medial temporal and frontal cortices, as well as the cerebellum. These effects may be related to the amount of cannabis exposure. Functional neuroimaging studies suggest different patterns of resting global and brain activity during the performance of several cognitive tasks both in adolescents and adults, which may indicate compensatory effects in response to chronic cannabis exposure.

**Limitations:**

However, the results pointed out methodological limitations of the work conducted to date and considerable heterogeneity in the findings.

**Conclusion:**

Chronic cannabis use may alter brain structure and function in adult and adolescent population. Further studies should consider the use of convergent methodology, prospective large samples involving adolescent to adulthood subjects, and data-sharing initiatives.

## Introduction

Cannabis is the illicit drug most widely available and used worldwide [1], [2], consumed by between 125 and 203 million people, largely younger age group (15–34 years), which corresponds to an annual prevalence rate of 2.8%–4.5% [1], [2]. Despite the fact that many individuals tend to discontinue cannabis use after their initial experimentation with the drug [1] and the percentage of individuals who develop dependence is lower than that associated with alcohol (15%) or tobacco (32%) use, around 9% of cannabis users develop dependence in the long term [3], [4]. Cannabis use has been associated with a range of acute and chronic mental health problems, such as anxiety, depression, neurocognitive alterations and deficits as well as increased risk of psychotic symptoms and disorders, the severity of these effects being dependent on frequency of use, age of onset and genetic vulnerability [5]–[15]. These effects are probably related to effects on the endocannabinoid system, which can modulate the neuronal activity of other neurotransmitter systems, such as dopamine, through its action on the most abundant cannabinoid receptor in brain, the cannabinoid receptor 1 (CB1) [16], [17]. CB1 receptors mature slowly, reaching maximal levels during adolescence [18], and are particularly concentrated in brain regions that are critical for executive functioning, reward processing and memory, such as the prefrontal cortex, anterior cingulate cortex, basal ganglia, medial temporal areas (e.g., hippocampus and amygdala) and cerebellum [19].

Animal studies have consistently demonstrated that delta-9-tetrahydrocannabinol (THC), the main psychoactive component of cannabis [20], is able to disrupt the regulatory role of the endogenous cannabinoid system [21], inducing neurotoxic changes in brain regions rich with cannabinoid receptors that might dramatically affect the process of maturational refinement of cortical neuronal networks [22]–[24] and lastly promote changes in brain structure and alter emotional and cognitive performance [25], particularly if the exposure has been during the adolescent period [26], [27]. In contrast to animal literature, results from human studies investigating chronic cannabis users are often inconsistent. These discrepancies may be due to heterogeneity in socio-demographic characteristics of the population studied, imaging techniques employed, as well as differences in drug usage patterns and psychiatric comorbidities that may not always be apparent or result in contact with mental health services and hence may not be appropriately controlled for in studies where participants are screened for presence of co-morbid psychiatric disorder merely by enquiring about previous contact with mental health services [28]–[30]. However, overall the results suggest that long-term cannabis use may result in persistent alterations in brain function and morphology that would extend beyond the period of intoxication [28], [31], and that earlier onset of use may be associated with greater detrimental effects [32], [33].

It is remarkable to note that although the onset of cannabis use is typically during adolescence, a few imaging studies have been conducted with adolescent users [28], [34]. Since brain development continues up to young adulthood [35], adolescence may be a critical period during which chronic cannabis exposure may have far-reaching consequences [36]. Although brain size is thought to stabilize around the age of five years [37], important neurodevelopmental processes continue throughout adolescence, including myelinization [38], synaptic refinement [39] and gray matter volume reduction [40]. While the long-term effects of cannabis use may potentially have major implications for social and family life, education and occupational functioning, its effects on brain structure and function have not been well determined.

The growing concern about cannabis use has led to a significant increase in the number of human studies using neuroimaging techniques to determine the effect of the substance on brain structure and function, as well as to several recent reviews examining this topic [28], [29], [34], [41]–[46]. However, some authors have only reviewed studies investigating the acute effects of cannabis [45], [46] or those published over the last decade [41], [44], while others did not adequately specify criteria for selecting studies [41], [43] or included those studies that investigated only adult population [29], [42]. In the present review, we have conducted a systematic literature search to assess and integrate the evidence of the impact of chronic cannabis use on brain structure and function, focusing on studies in the adolescent and adult population. Papers published until August 2012 have been included following a comprehensive search strategy and pre-determined set of criteria for article selection [29].

## Methods

Data for this systematic review was collected with an advanced document protocol in accordance with the PRISMA guidelines [47]. This protocol provided a checklist for reporting systematic reviews (see Table S1).

### Search strategy

Electronic searches were performed using EMBASE (1980-August 2012), Medline (1966-August 2012), PubMed (1966-August 2012) and LILACS (1982-August 2012) databases. The following key words were used: cannabis; marijuana; marihuana; delta-9-tetrahydrocannabinol; THC; cannabidiol, CBD; neuroimaging; brain imaging; computerized tomography, CT; magnetic resonance, MRI; single photon emission tomography, SPECT; functional magnetic resonance, fMRI; positron emission tomography, PET; diffusion tensor MRI, DTI-MRI; spectroscopy, MRS. All the studies published up to August 2012 were included without language restriction.

### Selection criteria

**Figure 1:**
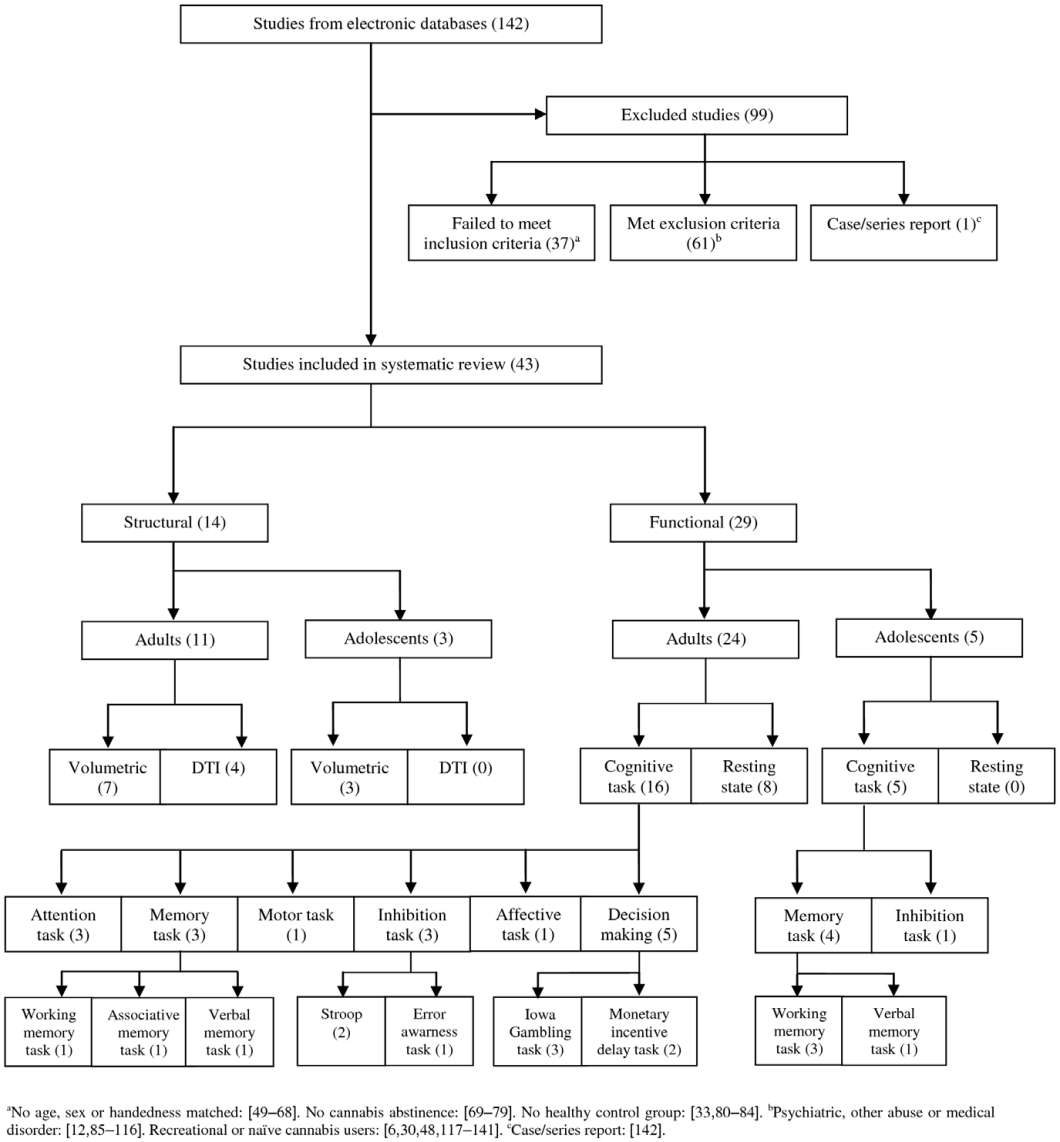
Flow diagram of included neuroimaging studies in chronic cannabis users.

A general review of all neuroimaging studies investigating brain structure or function was initially performed. We obtained a total of 142 published papers (Figure 1). Studies were included or excluded if they expressly stated the following criteria. Inclusion criteria were: (i) use of structural or functional neuroimaging techniques involving chronic cannabis users; (ii) inclusion of a control group of healthy volunteers matched by age, gender and handedness; and (iii) users had to be abstinent for at least 12 hours before brain scanning. Exclusion criteria were: (i) non-neuroimaging studies of cannabis use; (ii) neuroimaging studies that involved participants who had other neurological or psychiatric disorders, or individuals who met criteria for alcohol dependence or other substance use disorders (abuse or dependence) different from cannabis and nicotine, or participants who were not abstinent or who tested positive for drugs other than cannabis on urine screening test; and (iii) neuroimaging studies with recreational or naïve cannabis users.

We defined chronic cannabis users as persons who used cannabis several times a week and who had done so for at least two years. Recreational (or occasional) cannabis users were defined as persons who had used cannabis sporadically (less than four times a month), and naïve users or healthy controls were persons who had used cannabis less than 15 times in their lifetime, according to standardized strict criteria [29], [48].

Any publication that reported data using two different neuroimaging techniques from the same subjects (e.g., structural MRI and functional MRI) or a study examining the same subjects with two different cognitive tasks (e.g., verbal working memory and visual attention task) was considered as two studies in this review.

### Data extraction

Data was independently extracted by two reviewers. In case of disagreement, opinion from a third senior researcher was sought to assess whether study criteria were fulfilled. From the articles included we recorded names of authors, year of publication, socio-demographic (e.g., sample size, gender, age, handedness) and cannabis use characteristics (e.g., duration, age of onset, frequency of cannabis use), imaging type and design, exclusion criteria (for neurological, psychiatric or drug history), confirmation of abstinence from other drugs (whether checked by urine test), rest/active condition (for functional imaging studies), type of cognitive task performed during functional imaging and psychopathological variables assessed (e.g., psychotic or depressive symptoms). With regard to alcohol use, we assessed if subjects met criteria for alcohol abuse or for excessive alcohol consumption (more than 21 or 14 standard alcohol units per week for males or females, respectively) based on the reported data. For structural and functional imaging data, the primary measures of interest were global and regional volume, and global and regional activity [cerebral blood flow (CBF), regional CBF (rCBF) or blood oxygen level dependent signal BOLD)]. The secondary outcome was its correlation with clinical variables. We collected the statistically significant results of each outcome variable, and recorded whether a multiple comparison correction was done to prevent bias towards false positives.

## Results

Of the 142 studies identified, thirty-six did not meet the *a priori* selection criteria [33], [49]–[84] and sixty-two met the exclusion criteria [6], [12], [30], [48], [85]–[141] or were case/series reports [142] (for more detailed information, see Figure 1). The remaining 43 studies were classified according to the neuroimaging technique used (structural/functional), age of the participants [adolescents (≤ 18 years) and adults (> 18 years] and testing conditions (resting state/cognitive task) (Figure 1). The studies included comprised: 14 structural neuroimaging studies [11 in adult users and 3 in adolescent users; 10 volumetric studies and 4 diffusion tensor imaging studies (DTI)] and 29 functional neuroimaging studies on the chronic effects of cannabis (24 in adult users and 5 in adolescent users; 8 in the resting state and 21 during a cognitive task).

### 1. Structural neuroimaging studies in adult chronic cannabis users

We identified 11 structural MRI studies that examined adult chronic cannabis users and met our selection criteria (Table 1). Structural differences were obtained in seven of them in terms of global brain measures [143] or gray/white matter changes [144]–[149]. Four studies did not find any significant structural alterations when comparing chronic cannabis users with healthy controls [150]–[153]. The abstinence period for all participants before they underwent the structural MRI was between 12 and 24 hours, apart from two studies [145], [152] (for details see Table 1).

**Table 1:** Structural neuroimaging studies in chronic cannabis users.

Author (yr.)	Method	CU M/F	HCM/F	Mean (SD) age CU/HC	Image analysis	Abstinence (Mean days)	Results*				
**ADULTS**							Global measures	Regionalmeasures(GM)	Regional measures (WM)	Detailed results	Correlations with clinical variables
							GM-WM CSF-TIV	FL-PL-TL-OL BG-Cb			
Block *et al*. (2000) [143]	MRI 1.5T	9/9	7/6	22.3 (0.5)/ 22.6 (0.5)	Whole brainROI	≤ 1	○ ○ • ○	○ ○ ○ ○ ○ ○	○	↓ CSF	
Tzilos *et al*. (2005) [153]	MRI 1.5T	16/6	19/7	38.1 (6.2)/ 29.5 (8.5)	ROI	≤ 1	○ ○ ○ ○	○			
Matochik *et al*. (2005) [148]	MRI 1.5T	11/0	8/0	25.4 (5)/ 29.7 (4.7)	Whole brainROI†	≤ 1		• ○ • ○ • ○	•	GMd: ↓ R parahippocampus; ↑ precentral gyrus and R thalamus. Hippocampus (ROI): ↓ GMdWMd: ↓ L PL; ↑ parahippocampus and fusiform gyrus, lentiform nucleus and pons	↑ WMd L precentral gyrus with duration of use (yr.)
Gruber *et al*. (2005) [151]	DTI 3T	8/1	8/1	26 (3.6)/ 26.2 (3.1)	Whole brainROI†	NE			○		
DeLisi *et al*. (2006)** [150]	DTI 1.5T	9/1	9/1	21.1 (2.9)/ 23 (4.4)	Whole brainROI	≤ 1	○ ○ ○ ○	○	○		
Jager *et al*. (2007) [152]	MRI 1.5T	13/7	13/7	24.5 (5.2)/ 23.6 (3.9)	Whole brainROI†	7		○	○		
Yücel *et al*. (2008) [149]	MRI 3T	15/0	16/0	38.8 (8.9)/ 36.4 (9.8)	ROI	≤ 1	○ ○ ○	•		Hippocampus: ↓ L/RAmygdala: ↓ L/R	↓ L hippocampus with cumulative exposure (yr.) and higher positive psychotic symptoms
Arnone *et al*. (2008) [144]	DTI 1.5T	11/0	11/0	25.0 (2.9)/ 23.3 (2.9)	ROI	≤ 1			•	Corpus callosum: ↑ MD	
Ashtari *et al*. (2011)*** [145]	MRI 1.5T	14/0	14/0	19.3 (0.8)/ 18.5 (1.4)	ROI	201	○ ○ ○ ○	•		Hippocampus: ↓ L/R	↑ hippocampus with verbal learning and memory scores in HC; ↓ hippocampus with amount of cannabis use
Gruber *et al*. (2011) [147]	DTI 3T	14/1	14/1	25.0 (8.7)/ 25.2 (8.4)	ROI	≤ 1			•	R Genu: ↑ MDL FL: ↓ FA	↑ FA L frontal with higher BIS total and motor subscale score; ↑ FA and ↓ MD in FL with later age of onset
Cousijn *et al*. (2011) [146]	MRI 3T	21/12	26/16	21.3 (2.4)/ 21.9 (2.4)	Whole brainROI†	≤ 1	○ ○ ○ ○	○ ○ • ○ ○ •	○	GM: ↑ anterior cerebellum	↓ R amygdala and ↓ L/R hippocampus with amount of cannabis use (weekly) or severity of cannabis dependence
**ADOLESCENTS**											
Medina *et al*. (2009)† [154]	MRI 1.5T	12/4	10/6	18.1/ 17.9 (16–18.9)	ROI	28	○	•	○	PFC: ↑ in F CUPFC: ↓ in M CU	↓ PFC in CU and ↑ PFC in HC with better executive functioning
Medina *et al*. (2010)† [155]	MRI 1.5T	12/4	10/6	18 (0.7)/ 18 (0.9)	ROI	28	○	•		Cerebellum: ↑ inferior posterior (lobules VIII–X) vermis	↑ Vermis with poorer executive functioning
McQueeny *et al*. (2011) [156]	MRI 3T	27/8	36/11	18.0/ 17.7	ROI	28	○	•		R amygdala: ↑ in F CU	↑ R amygdala with internalizing symptoms in F CU

Note: Yr.  =  Years; CU  =  Cannabis users; HC  =  Healthy controls; M  =  Male; F  =  Female; SD  =  Standard deviation; GM  =  Grey matter; GMd  =  Grey matter density; WM  =  White matter; WMd  =  White matter density; CSF  =  Cerebral spinal fluid; TIV  =  Total intracranial volume; FL  =  Frontal lobe; PL  =  Parietal lobe; TL  =  Temporal lobe; OL  =  Occipital lobe; BG  =  Basal ganglia; Cb  =  Cerebellum; L  =  Left hemisphere; R  =  Right hemisphere; T  =  Tesla; MRI  =  Magnetic resonance imaging; DTI  =  Diffusion tensor imaging; ROI  =  Region of interest; NE  =  Not specified; MD  =  Mean diffusivity; FA  =  Fractional anisotropy; PFC  =  Prefrontal cortex; BIS  =  Barrat Impulsivity scale.

* If not otherwise specified, results are presented in terms of chronic cannabis users.

•  =  Significant differences; ○  =  Non-significant differences;  =  Not examined.

** Two subjects in the marijuana group met criteria for excessive alcohol consumption.

*** Five subjects in the marijuana group met criteria for alcohol abuse.

† Subjects with symptoms of alcohol abuse or dependence were included.

† Multiple comparison correction.

#### 1.1. Volumetric studies

Of the seven studies comparing global brain volume measures between chronic cannabis users and healthy controls, there was only one study reporting significant differences [143], namely reduced ventricular cerebral spinal fluid (CSF) in cannabis users. Another study [145] reported total brain volume difference between groups which was no longer significant when the authors covaried for confounding factors such as premorbid intelligence.

Among the six studies employing a whole-brain analysis approach [143], [146], [148], [150]–[152], two further studies described differences between chronic cannabis users and controls [146], [148]. Matochik *et al*. (2005) [148] found lower grey matter density in the right parahippocampus and greater grey matter density in the precentral gyrus and right thalamus in cannabis users, while Cousjin *et al*. (2011) [146] found a larger anterior cerebellum in cannabis users. Matochik *et al*. (2005) [148] also reported differences in white matter density, such as lower density in the left parietal lobe and higher in parahippocampus, fusiform gyrus, lentiform nucleus and pons.

With regard to the three studies that focused on specific regions of interest, all studies reported bilateral volumetric reductions in the hippocampus [145], [148], [149] and one reported volume reductions in the right amygdala [149]. Some studies have also reported correlations between regional brain volume measures and cannabis use parameters, clinical and neuropsychological measures. For instance, a smaller hippocampal volume has been related to a greater exposure to cannabis [145], [146], [149], severity of cannabis dependence [146] and more severe positive psychotic symptoms [149]. Ashtari et al. (2011) [145] described a positive association between larger hippocampus volumes and higher verbal learning and memory scores in healthy controls but not in cannabis users [145]. It is remarkable to note that these findings were in patients with an average of 6.7 months of abstinence, which appears to support of the idea that cannabis use may cause long-term brain alterations.

With respect to other brain regions, Cousijn *et al*. (2011) [146] reported a negative correlation between amygdala volume and the amount of cannabis use or dependence, while Matochik *et al*. (2005) [148] found an association between increased white matter density in left precentral gyrus and longer duration of cannabis use.

#### 1.2. Diffusion tensor imaging (DTI) studies

Four studies have used DTI to examine the integrity of white matter tracts in chronic cannabis users [144], [147], [150], [151], of which half have reported positive results [144], [147]. Arnone *et al*. (2008) [144] found increased mean diffusivity (MD) in the corpus callosum while Gruber *et al*. (2011) [147] found increased MD in the right genu as well as reductions in left frontal fractional anisotropy (FA). Gruber *et al*. (2011) [147] also reported a positive association between left frontal FA and impulsivity scores, and higher FA and lower MD in the frontal lobes being associated with a later age of initiation of cannabis use.

### 2. Structural neuroimaging studies in adolescent chronic cannabis users

Three volumetric studies in adolescent chronic cannabis users were included, two of which consist of the same sample [154], [155]. As an exception, these two studies [154], [155] were included despite involving participants with symptoms of alcohol dependence given the modest number of studies included in this population (for details see Table 1). The MRI scans, focused on specific regions of interest and were obtained following 28 days of abstinence from cannabis use. Medina *et al*. (2009, 2010) [154], [155] reported significantly larger volumes of the inferior posterior vermis, as well as a marginal group-by-gender interaction in the prefrontal cortex, in which female and male cannabis users demonstrated, respectively, larger and smaller prefrontal cortex volumes compared to the same-gender controls. McQueeny *et al*. (2011) [156] also described an effect of gender in which female cannabis users but not males, exhibited a larger right amygdala volume.

In terms of correlations, Medina *et al*. (2010) [155] found that larger volumes of the vermis were associated with poorer executive functioning while McQueeny *et al*. (2011) [156] found that larger right amygdala volume was associated with more internalizing symptoms (e.g., anxiety/depression). Lastly, Medina *et al*. (2009) [154] also found that increased volume in the prefrontal cortex was associated with poorer executive functioning among cannabis users while the opposite pattern was observed in controls, suggesting that female users may be at increased risk for cannabis-induced prefrontal abnormalities.

### 3. Functional neuroimaging studies in adult chronic cannabis users

#### 3.1. Resting state

We included eight case-control studies comparing resting rCBF in adult chronic cannabis users and non cannabis using healthy controls (Table 2). The imaging methods used were as follows: H2^15^O-PET [157], ^133^Xe-SPECT [158], ^18^F-FDG-PET [159], [^11^C]- raclopride-PET [159]–[162] and [^18^F]FMPEP-d2 [163]. Functional differences between groups were found in all studies, except for the four [^11^C]-raclopride-PET studies [159]–[162]. Abstinence periods ranged from 12 hours to 542 days (for details see Table 2). Block *et al*. (2000) [157] described reduced bilateral rCBF in the posterior cerebellum and ventral prefrontal cortex but also increased rCBF in the anterior cingulate cortex in cannabis users. Lundqvist *et al*. (2001) [158] found a trend of lower global CBF in cannabis users, as well as reduced rCBF in the right prefrontal and superior frontal cortex. Sevy *et al*. (2008) [159] reported lower glucose metabolism in the right orbitofrontal cortex, putamen bilaterally and precuneus in chronic cannabis users. However, there were no significant differences between the groups in striatal D2/D3 receptor availability and no correlation between striatal [^11^C]-raclopride-PET binding potential and glucose metabolism [159]. Consistent with these results, three other [^11^C]- raclopride-PET studies [160]–[162] failed to find any differences between groups in dopamine D2/D3 receptor availability in the striatum as a whole or it functional subdivisions. However, while Stokes *et al*. (2012) [160] also failed to find any association between lifetime frequency of cannabis use and binding potential values, Albrecht *et al*. (2012) [161] described a negative correlation with both urine levels of cannabis metabolites and self-report of recent cannabis consumption. Finally, Hirvonen *et al*. (2011) [163] demonstrated a reversible and regionally selective downregulation of CB1 receptors. At baseline, current users had approximately 20% less CB1 receptor density in the neocortex and limbic regions, which was negatively correlated with years of cannabis exposure. After four weeks of abstinence from cannabis use, CB1 receptor density returned to normal levels in all brain regions, except for the hippocampus [163].

**Table 2:** Functional neuroimaging studies in chronic cannabis users.

Author (yr.)	Method	CUM/F	HCM/F	Mean (SD) age CU/HC	Image analysis	Condition	Abstinence (Mean days)	Results*		
**ADULTS**								Brain area	Detailed results	Correlations with clinical variables
**Functional (resting state)**								FL-PL-TL-OL I-BG-Cb		
Block *et al*. (2000) [157]	H_2_^15^O-PET	8/9	6/6	22.4 (0.5)/22.6 (0.5)	Whole brain	Resting state	≤ 1	• ○ ○ ○ ○ ○ •	↓ rCBF L/R cerebellum and VMPFC↑ rCBF R ACC	
Lundqvist *et al*. (2001) [158]	^133^Xe-SPECT	12/0	14/0	29.8 (5)/ 27.8 (5.2)	Whole brain	Resting state	1.6	• ○ ○ ○ ○ ○ ○	↓ global CBF (trend)↓ rCBF R superior PFC, superior frontal	
Sevy *et al*. (2008) [159]	^18^F-FDG-PET	6/0	6/0	20.0 (1.0)/20.0 (1.0)	Whole brain	Resting state	105	• • ○ ○ ○ • ○	↓ rCBF R OFC and R posterior parietal cortex and L/R putamen	
Sevy *et al*. (2008) [159]	[^11^C]-raclopride-PET	6/0	6/0	20.0 (1.0)/20.0 (1.0)	ROI	Resting state	105	○		
Hirvonen *et al*. (2011) [163]	[^18^F]FMPEP-d_2_	30/0	28/0	28 (8)/ 32 (10)	Whole brainROI	Resting state	1 and 26	• • • • ○ ○ ○	1 day: ↓ V_T_ neocortex and limbic cortex26 days: ↑ V_T_ neocortex and limbic cortex except for hippocampus	↓ V_T_ with longer cannabis exposure (yr.)
Stokes *et al*. (2011) [160]	[^11^C]-raclopride-PET	6/4	9/1	32.6 (7.7)/ 36.5 (4.5)	ROI	Resting state	542	○		
Urban *et al*. (2012) [162]	[^11^C]-raclopride-PET	15/1	14/2	27.3 (6.1)/ 28.1 (6.9)	ROI	Resting state	30	○		
Albrecht *et al*. (2012) [161]	[^11^C]-raclopride-PET	10/0	8/0	25.1 (4.6)/ 26.4 (5.6)	ROI	Resting state	≤ 1	○		↓ BP_ND_ with increase in urine levels of THC-COOH and self-reported recent intake per day
**Functional (cognitive task)**										
Block et al. (2002) [164]	H_2_^15^O-PET	18/0	13/0	22.3 (0.5)/ 22.6 (0.5)	Whole brainROI	Verbal memory	≤ 1	• ○ • ○ ○ ○ •	↓ rCBF L/R PFC↑ rCBF L > R hippocampus in HC↑ rCBF posterior cerebellum	
Eldreth et al. (2004) [166]	H_2_^15^O-PET	11/0	11/0	25/29	Whole brainROI†	Stroop	25	• ○ • • ○ ○ ○	↑ CBF R paracentral lobule and L occipital lobe↓ CBF R VMPFC and R DLPFCHippocampus: ↑ rCBF L/RACC:↓ rCBF LDLPF: ↑ rCBF L/R	
Bolla et al. (2005) [165]	H_2_^15^O-PET	11/0	11/0	26 (21–35)/ 31	Whole brainROI†	Iowa Gambling	25	• ○ • • ○ ○ •	↓ CBF OFC and R middle frontal gyrus↑ CBF L cerebellum, R inferior frontal gyrus and R occipital lobe in moderate CU↑ CBF L cerebellum in heavy CUCerebellum: ↑ rCBF LOFC: ↓ rCBF RDLPFC: ↓ rCBF R	↓ rCBF R cerebellum, R orbital gyrus and ↑rCBF R parahippocampus with cannabis exposure
Gruber et al. (2005) [151]	fMRI 3T	8/1	8/1	26 (3.6)/ 26.2 (3.1)	ROI†	Stroop	NE	•	ACC: ↓ BOLD L/RDLPFC: ↑ BOLD R	
Chang et al. (2006) [169]	fMRI 4T	15/9	11/8	27.9 (10.8)/ 30.6 (8.0)	Whole brainROI†	Visual attention task	≤ 1 and 1157	• • ○ • ○ ○ •	↓ BOLD L/R PFC, R medial and dorsal parietal cortex and cerebellum↑ BOLD frontal, parietal and occipital lobules↑ BOLD cerebellum in current CU	↑ BOLD R PFC and dorsal parietal cortex and ↓ BOLD cerebellum with age of cannabis onset; ↓ BOLD cerebellum with cannabis exposure; ↑ BOLD R PFC and cerebellum (normalization) with duration of cannabis abstinence
Jager et al. (2006) [173]	fMRI 1.5T	7/3	7/3	22.7 (4.2)/ 22.8 (2.9)	Whole brainROI†	Working memory	7	○ • ○ ○ ○ ○ ○	↑ BOLD L superior parietal cortex	
Jager et al. (2007) [152]	fMRI 1.5T	13/7	13/7	24.5 (5.2)/ 23.6 (3.9)	Whole brainROI†	Associative memory	7	• ○ • ○ ○ ○ ○	↓ BOLD R DLPFC and L/R parahippocampusParahippocampus: ↓ BOLD L/R	
Hester *el al*. (2009) [172]	fMRI 3T	15/1	15/1	24.6 (1.5)/ 25.2 (1.3)	Whole brainROI†	Error Awareness Task	≤ 1	• • ○ ○ • ○ ○	↓ BOLD ACC, R insula, L/R inferior parietal and middle frontal regions	
Gruber *et al*. (2009) [170]	fMRI 3T	14/1	14/1	25 (8.8)/ 26 (9.0)	ROI	Masked affective stimuli	≤ 1	• •	ACC: ↑ BOLD (masked happy faces) and ↓ BOLD (masked angry stimuli)Amygdala: ↓ BOLD (masked angry stimuli)	↑ BOLD ACC with cannabis exposure (per week) in masked angry faces and with cannabinoid level in masked happy faces; ↑ BOLD amygdala with cannabis exposure (per week) in masked happy faces
van Hell *et al*. (2010) [176]	fMRI 1.5T	14/1	11/2	24 (4.4)/ 24 (2.7)	Whole brainROI†	Monetary incentive delay task	7	• ○ • • ○ • ○	Compared to non-smoking HC: ↓ BOLD NAcc, caudate nucleus, L putamen, R inferior and medial frontal gyrus, L/R superior frontal gyrus, L cingulate gyrus; ↑ BOLD L/R temporal gyrus, R cuneus and R parahippocampus gyrusCompared to smoking HC: ↓ BOLD caudate nucleus, L/R superior frontal; ↑ BOLD L middle temporal gyrus	
Nestor *et al*. (2010) [175]	fMRI 3T	11/3	12/2	22.1 (1.2)/ 23.1 (1.2)	Whole brain	Monetary incentive delay task	4.5	○ ○ ○ ○ • • ○	↑ BOLD R VS (win cue periods)↓ BOLD L insula (after loss and loss avoidance)	↑ BOLD R VS (win cue periods) with cannabis exposure (yr.)
Abdullaev *et al*. (2010) [168]	fMRI 3T	10/4	10/4	19.5 (0.8)/ 19.7 (1.4)	Whole brain†	Attention task	2	• • ○ ○ ○ ○ ○	↑ BOLD R PFC and L/R parietal cortex	
Wesley *et al*. (2011) [177]	fMRI 1.5T	9/7	6/10	26.4 (3.6)/ 26.6 (6.1)	Whole brain	Iowa Gambling	≤ 1	• • ○ • ○ ○ •	↓ BOLD ACC, VMPFC and medial frontal cortex, precuneus, superior parietal, occipital and cerebellum	BOLD activity in ACC, VMPFC and rostral PFC predicted improvement over the task course only in HC
King *et al*. (2011) [174]	fMRI 3T	16/14	16/14	23.7/21.7	ROI	Finger sequencing	≤ 1	• •	Lingual gyrus: ↓ BOLDSuperior frontal gyrus: ↑ BOLD	↑ BOLD superior frontal gyrus with cortisol levels in F CU
Vaidya *et al*. (2012) [167]	H_2_^15^O-PET	28/18	18/16	24.3 (3.9)/ 24.7 (5.3)	Whole brain	Iowa Gambling	≤ 1	• ○ ○ ○ • ○ •	↑ rCBF VMPFC and cerebellumVariant IGT: ↑ rCBF VMPFC, cerebellum and anterior insula	↑ rCBF VMPFC with duration of cannabis use
Harding *et al*. (2012) [171]	fMRI 3T	11/10	10/11	36.5 (8.8)/ 31 (11.7)	ROI	MSIT	≤ 1	• ○ • • • ○ ○	↑ connectivity between ACC, L PFC, L/R anterior insula and the L occipitoparietal cortex	↑ connectivity with age of onset and lifetime exposure to cannabis
**ADOLESCENTS**										
**Functional (cognitive task)**										
Tapert *et al*. (2007) [183]	fMRI 1.5T	12/4	12/5	18.1 (0.7)/ 17.9 (1.0)	Whole brain	Response inhibition task	28	• • ○ • • ○ ○	↑ BOLD R DLPFC, L/R medial frontal and inferior and superior parietal and R occipital gyrus↑ BOLD R PFC, insular and parietal cortex during go trials	
Padula *et al*. (2007)** [179]	fMRI 1.5T	14/3	12/5	18.1 (0.8)/ 17.9 (1.1)	Whole brain	Spatial working memory	28	• • • ○ ○ • ○	↑ BOLD R basal ganglia, R precuneus, postcentral gyrus and L/R superior parietal cortex	↑ BOLD L temporal gyrus and L ACC (↓ in HC) and ↓ BOLD R temporal gyrus, R thalamus and pulvinar, L parahippocampus (↑ in HC) with higher score on SWM task
Schweinsburg *et al*. (2008)*** [180]	fMRI 1.5T	11/4	12/5	18.1 (0.7)/ 17.9 (1.0)	Whole brain	Spatial working memory	28	• • ○ • ○ ○ ○	↓ BOLD R DLPFC and occipital cortex↑ BOLD R posterior parietal cortex	
Schweinsburg *et al*. (2010)† [181]	fMRI 1.5T	9/4 – 9/4	11/7	17.1 (0.5)-17.6 (0.9)/17.3 (0.8)	Whole brain†	Spatial working memory	3 and 38	• ○ ○ ○ • ○ ○	↑ BOLD L superior PFC and L/R anterior insula in recent CU↑ BOLD R precentral gyrus in abstinent CU	
Schweinsburg *et al*. (2011) [182]	fMRI 3T	4/4	16/6	18.1 (0.9)/ 17.6 (0.8)	Whole brainROI	Verbal paired associates task	25	○ ○ ○ ○ ○ ○ ○		

Note: Yr.  =  years; CU  =  Cannabis users; HC  =  Healthy controls; M  =  Male; F  =  Female; SD  =  Standard deviation; FL  =  Frontal lobe; PL  =  Parietal lobe; TL  =  Temporal lobe; OL  =  Occipital lobe; I  =  Insula; BG  =  Basal ganglia; Cb  =  Cerebellum; fMRI  =  Functional magnetic resonance imaging; SPECT  =  Single photon emission tomography; PET  =  Positron emission tomopgraphy; DSC  =  Dynamic susceptibility contrast; V_T_  =  Distribution volume; BP_ND_  =  Non-displaceable binding potential; FDG  =  Fludeoxyglucose; L  =  Left hemisphere; R  =  Right hemisphere; ROI  =  Region of interest; MSIT  =  Multi-Source Interference Task; CBF  =  Global cerebral blood flow; rCBF  =  Regional cerebral blood flow; BOLD  =  blood oxygenation-level dependent; NE  =  Not specified; PFC  =  Prefrontal cortex; DLPFC  =  Dorsolateral prefrontal cortex; VMPFC  =  Ventromedial prefrontal cortex; OFC  =  Orbitofrontal cortex; ACC  =  Anterior cingulated cortex; NAcc  =  Nucleus accumbens; VS  =  Ventral striatum; STG  =  Superior temporal gyrus; SWM  =  Spatial working task; IGT  =  Iowa Gambling Task.

* If not otherwise specified, results are presented in terms of chronic cannabis users.

○  =  Significant differences; ○  =  Non-significant differences;  =  Not examined.

** Two subjects in the marijuana group met criteria for alcohol abuse.

*** Four teens in the chronic cannabis group met criteria for alcohol use disorder, two cases of abuse and two cases of dependence.

† One control, three recent users and two abstinent users met criteria for alcohol use disorders.

† Multiple comparison correction.

#### 3.2. Cognitive paradigms

We identified 16 studies in adult chronic cannabis users that compared regional activation during the performance of a cognitive task with healthy controls (Table 2), four with PET [164]–[167] and twelve with fMRI [151], [152], [168]–[177].

### Attention

Chang *et al*. (2006) [169] used fMRI to compare a visual-attention task in current and abstinent cannabis users with healthy controls. Despite all groups showing normal task performance, both active and abstinent chronic cannabis users demonstrated decreased activation in the right prefrontal, medial and dorsal parietal cortices and medial cerebellar regions but greater activation in several smaller regions throughout the frontal, posterior parietal, occipital and cerebellum. An apparent normalization of BOLD signal was described in the right prefrontal and medial cerebellar regions in those with a longer duration of abstinence. In addition, early age of onset and estimated cumulative cannabis lifetime exposure were both associated with reduced activation in the right prefrontal cortex and medial cerebellum. More recently, Abdullaev *et al*. (2010) [168] used two attention tasks [the use generation task and the attention network task (ANT)] to contrast differences between cannabis users and healthy controls. Chronic cannabis users showed poorer performance in the ANT (more errors and longer reaction time), as well as stronger activation within the right prefrontal cortex in both tasks and within the parietal cortices in the ANT, which may indicate a less efficient system for the executive control of attention during conflict resolution tasks. Finally, Harding *et al*. (2012) [171] demonstrated for the first time that long-term heavy cannabis use is associated with increased functional connectivity between several frontal cortex regions and the occipitoparietal cortex using the Multi-Source Interference Task (MSIT). No differences in behavioural performance were evident between groups. The authors suggest that their findings may suggest a compensatory role for these regions in mitigating the effects of abnormal attentional and visual processing following chronic cannabis exposure [171].

### Memory

In a H2^15^O-PET study, Block *et al*. (2002) [164] found that cannabis users performed verbal memory tasks more poorly than controls. This was associated with reduced activation in the prefrontal cortex and greater activation in the posterior cerebellum, as well as with an absence of lateralization of hippocampal activity. Consistent with this, Jager *et al*. (2007) [152] described attenuated activity in the right dorsolateral prefrontal cortex and bilateral (para) hippocampal gyri in cannabis users despite normal performance in an associative memory task. Finally, in a verbal working memory task, Jager *et al*. (2006) [173] found significantly greater activity in the left superior parietal cortex in the cannabis using group despite there being no differences in task performance, which may be consistent with the idea of a compensatory recruitment effect.

### Inhibition and impulsivity

Eldreth *et al*. (2004) [166] and Gruber *et al*. (2005) [151] studied the degree of inhibitory control during a Stroop task in current (positive THC urine analysis) and abstinent chronic cannabis users, respectively. Gruber *et al*. (2005) [151] found lower anterior cingulate activity and higher mid-cingulate and bilateral dorsolateral prefrontal cortex activity in current cannabis users relative to healthy controls, who demonstrated focal increased activity within the right dorsolateral prefrontal cortex. Consistently, Eldreth *et al*. (2004) [166] found in abstinent cannabis users a reduced anterior cingulate activation using H2^15^O-PET during the performance of a modified Stroop test. However, they also reported a reduced dorsolateral prefrontal cortex activation and a greater activation in the hippocampus bilaterally [166]. Lastly, Hester *et al*. (2009) [172] administered a go/no-go response inhibition task to active cannabis users to determine inhibitory control and error awareness compared with healthy controls. Although control performance was equivalent between the two groups, cannabis users displayed a significant deficit in awareness of commission errors, which was associated with decreased a activity in the anterior cingulate cortex and right insula, as well as in the bilateral inferior parietal and middle frontal regions [172].

### Decision-making

Bolla *et al*. (2005) [165] and Vaidya *et al*. (2011) [167] using H2^15^O-PET, and Wesley *et al*. (2011) [177] using fMRI, studied the brain activation pattern in chronic cannabis users compared to healthy controls during the Iowa Gambling Task (IGT). Bolla *et al*. (2005) [165] reported dysfunction during the performance of the task in abstinent cannabis users, demonstrating a lower activation in the right orbitofrontal cortex and dorsolateral prefrontal cortex and greater activation in the left parietal and cerebellar cortices. The number of joints used per week was positively correlated with activation in the right parahippocampal gyrus but inversely correlated with activation in the right cerebellum and orbital gyrus. Wesley *et al*. (2011) [177] also reported a poorer performance on the IGT in active cannabis users. However, there were no differences during the initial strategy development phase, in which cannabis users showed reduced activity in response to losses in anterior cingulate cortex, ventromedial prefrontal cortex, precuneus, superior parietal lobe, occipital lobe and cerebellum compared to controls [177]. Additionally, the functional response to losses in anterior cingulate, ventromedial and rostral prefrontal cortices was positively correlated with improvement over the task course only in the control group, indicating that cannabis users may be less sensitive to negative feedback during the strategy development phase [177]. In contrast, Vaidya *et al*. (2011) [167] did not find differences on the standard IGT performance between active cannabis users and healthy controls. Nevertheless, cannabis users performed significantly worse than controls on a variant version of the same task [178]. Both groups showed increased activity in ventromedial prefrontal cortex on both versions of the IGT compared to the control task but in contrast to Wesley *et al*. (2011) [177], cannabis users demonstrated greater activity than controls in the ventromedial prefrontal cortex on the standard IGT, as well as in the cerebellum and the anterior insula on both versions of the IGT [167]. Furthermore, duration of cannabis use was associated with greater activity in ventromedial prefrontal cortex [167]. Nestor *et al*. (2010) [175] and van Hell *et al*. (2010) [176] used fMRI to measure brain activity during reward and anticipation of loss with different versions of a monetary reward task. There were no significant behavioural differences between the groups in both studies. Nestor *et al*. (2010) [175] reported a greater right ventral striatum activity in cannabis users during reward anticipation, which was significantly correlated with years of lifetime cannabis use. In addition, response to loss and loss avoidance outcome notification was related with hypoactivity in left insula, and in the post hoc analysis comparing loss and win cues with no-outcome cues, right ventral putamen showed greater BOLD response [175]. Conversely, comparing cannabis users to non tobacco-smoking controls, van Hell *et al*. (2010) [176] demonstrated attenuated activity in the nucleus accumbens and caudate nucleus bilaterally during reward anticipation, as well as left putamen and right inferior and medial frontal gyrus, superior frontal gyrus bilaterally and left cingulate gyrus. Cannabis users showed enhanced reward anticipation activity in the middle temporal gyrus bilaterally, right cuneus and right parahippocampal gyrus. When compared to tobacco-smoking controls, cannabis users also showed reduced anticipation activity in the same areas, with the exception of the nucleus accumbens bilaterally, the right medial frontal gyrus and the left cingulated gyrus, indicating that anticipation activity in these regions may be attenuated by both cannabis and nicotine [176]. In accordance with Nestor *et al*. (2010) [175], response to contrasted outcome notification was associated with greater activity in the putamen bilaterally and the right caudate nucleus compared with non-smoking controls [176]. The putamen was more activated in cannabis users than in non-smokers and tobacco-smoking controls, indicating that changes in this area were mainly due to cannabis use [176].

### Motor performance

King *et al*. (2011) [174] reported that chronic cannabis use was associated with slower and less efficient psychomotor function, especially in male users. Cannabis users showed lesser activation in the lingual gyrus and greater activation of the superior frontal gyrus compared to controls while performing a visually paced finger sequencing task, suggesting that the former group shifted from more automated visually-guided responses to more executive or attention control regions of the brain [174].

### Affective processing

Gruber *et al*. (2009) [170] examined the BOLD signal changes for two target affective conditions (happy and anger). Region of interest analyses revealed that cannabis users demonstrated relatively lower anterior cingulate and amygdalar activity during the presentation of masked angry stimuli sets relative to the control group, who showed relatively higher activation within these regions. In contrast, cannabis users demonstrated a larger pattern of activation during the presentation of masked happy faces within the cingulate as compared to controls, with no increase in amygdalar activation [170]. Furthermore, the total number of smoking episodes per week was positively associated with cingulate activity during the viewing of masked angry faces and positively associated with amygdalar activity during the viewing of masked happy faces [170]. Finally, overall cannabinoid level was positively related to cingulate activity during the viewing of masked happy faces [170]. The disparate activation patterns showed between groups suggest a different way of processing emotional information between groups [170].

### 4. Functional neuroimaging studies in adolescent chronic cannabis

We included five case-control fMRI studies in adolescent cannabis users comparing brain activity with healthy controls during a cognitive task performance. As an exception, two of them [180], [181] were included despite involving a minor proportion of participants with a co-morbid alcohol dependence given the relatively modest number of studies in this population (for details see Table 2). No resting state studies were identified in the adolescent population.

### Memory

Padula *et al*. (2007) [179] and Schweinsburg *et al*. (2008, 2010) [180], [181] examined fMRI response during a spatial working memory (SWM) task. In a group of abstinent adolescent cannabis users, Padula *et al*. (2007) [179] described increased activity in the left temporal gyrus and anterior cingulate cortex but lower activity in right temporal gyrus, thalamus, pulvinar and left parahippocampal gyrus related to higher scores on the task, while the reverse pattern was found in the controls. This may suggest that cannabis users employed more of a verbal strategy to achieve the same level of task performance as the controls [179]. Additionally, cannabis users demonstrated greater performance-related activation in the right basal ganglia, precuneus, postcentral gyrus and bilateral superior parietal lobe [179], again suggesting a compensatory neural effort. Consistent with this, Schweinsburg *et al*. (2008) [180] also found a different pattern of activation in abstinent adolescent cannabis users who performed the SWM task similarly to the control group. Thus, cannabis users demonstrated higher activation in the right parietal cortex but also lower activity in the right dorsolateral prefrontal and occipital cortices [180]. Finally, in a cross-sectional study, Schweinsburg *et al*. (2010) [181] compared fMRI responses using the same task among adolescent cannabis users with brief and sustained cannabis abstinence and healthy controls. Although both groups performed at a similar level on the task, recent users showed greater activity in the medial and left superior prefrontal cortices and bilateral insula while abstinent users demonstrated an increased response in the right precentral gyrus [181]. More recently, Schweinsburg *et al*. (2011) [182] compared fMRI response during a verbal paired associates encoding task in 3 groups of participants that included an abstinent cannabis user group, a binge drinker group and a cannabis user group with co-morbid binge-drinking to healthy controls with very limited alcohol or cannabis experience. In general, each group displayed deviations in BOLD response relative to non-using controls, and binge drinking and cannabis use demonstrated independent as well as interactive effects on brain functioning [182].

### Inhibition and impulsivity

In a group of abstinent cannabis users, Tapert *et al*. (2007) [183] compared the activation pattern on a go/no-go task during fMRI with seventeen healthy subjects. Despite similar level of task performance, cannabis users showed greater activation during inhibitory trials in the right dorsolateral prefrontal, bilateral medial frontal, bilateral inferior and superior parietal lobules and right occipital gyrus compared to the healthy subjects. During the non-inhibitory trials, differences were located in right prefrontal, insular and parietal cortices, with cannabis users showing greater activation in these areas compared to the controls. As observed in adults, these results suggest a greater neurocognitive effort during the task in cannabis users, even after the abstinence period.

## Discussion

In this systematic review, we identified 43 studies suitable for inclusion regarding the impact of chronic cannabis use on brain structure and functioning, of which eight (19%) were in the adolescent population. Despite the high degree of heterogeneity among the studies reviewed herein, several relatively consistent findings emerged from this review. These findings, discussed in detail below, include: (1) Structural brain abnormalities, mainly in CB_1_-rich areas implicated in several cognitive functions, which may be related to the amount of cannabis use; (2) Altered neural activity during resting state and under several different types of cognitive paradigms, that may reflect a different recruitment of brain areas during the tasks, particularly within the prefrontal cortex; and (3) The few studies conducted in adolescents suggest that both structural and functional alterations may appear soon after starting the drug use and may be related to gender.

In terms of structural findings, specific regional brain analyses demonstrated evidence of structural abnormalities when adult chronic cannabis users were compared with healthy controls. The most consistently reported brain alteration was reduced hippocampal volume [145], [146], [148], [149], which was shown to persist even after several months of abstinence in one study [145] and also to be related to the amount of cannabis use [145], [146], [149]. Other frequently reported morphological brain alterations related to chronic cannabis use were reported in the amygdala [146], [149], [156], the cerebellum [146], [155] and the frontal cortex [148], [154]. Lastly, two DTI studies found differences in the mean diffusivity or fractional anisotropy in the corpus callosum and the frontal white matter fibre tract [144], [147], suggesting that chronic cannabis exposure may also alter white matter structural integrity, by either affecting demyelination or causing axonal damage or indirectly through delaying normal brain development. With regard to the few structural MRI studies focusing on the effects of cannabis use on brain morphology in adolescents, some discrepancies were reported related to adult population. These inconsistencies may be explained in terms of the disruption of normal pruning during developmental maturation due to early chronic cannabis use, ultimately resulting in larger regional volumes [156]. Notwithstanding, structural results from adolescent population suggest that the effects of chronic cannabis use may appear soon after starting the drug use, persist after a month of abstinence or even be moderated by gender [145], [154]–[156]. In this context, it has been reported that adolescent female cannabis users may be at increased risk for cannabis-induced morphological effects [154], [156].

Functional neuroimaging studies that have evaluated the resting state in active and abstinent adult chronic cannabis users suggest that resting global [158], prefrontal cortical [157]–[159], cerebellar [157] and striatal [159] blood flow may be lower compared with controls. These brain regions correspond to areas with relatively high concentration of CB1 receptors [19]. Hence, it has been hypothesised that the decreased resting state function may represent a down-regulation of CB1 receptors as a result of regular exposure to cannabis [41]. However, it is important to note that not all studies have consistently demonstrated effects in these regions. Furthermore, it has been recently found that, similar to animal studies, down-regulation of CB1 receptors in humans is region-specific and reversible, occurring in the neocortex and limbic cortex but neither in subcortical brain regions nor in the cerebellum [163]. It is also noteworthy that these brain regions correspond to areas that are engaged in the processing of reward [184]. This is also consistent with the evidence of neuropsychological impairments in chronic cannabis users, such as in attention and working memory [185], decision making [186], and psychomotor speed [187]. Also, consistent with experimental animal studies, no differences in striatal D2/D3 receptor availability were found in four studies of chronic cannabis users compared with healthy controls [159]–[162]. However, in the only study where the chronic cannabis users were not abstinent [161], an inverse correlation between recent cannabis consumption and D2/D3 receptor availability was found, leading the authors to suggest that this effect could be related to a direct effect of cannabis smoking on the expression of striatal DA receptors in heavy cannabis users [161]. Additional studies are needed to better understand the neurochemical basis of this finding.

Functional imaging studies comparing activation in both adult and adolescent chronic cannabis users with healthy controls during the performance of different cognitive tasks indicated that chronic cannabis users would use similar brain areas that engage these cognitive processes but often demonstrating an altered pattern of brain activity [151], [152], [157], [165]–[177], [179], [181]–[183]. However, the level of performance of the cannabis users on the cognitive tasks employed was generally similar to that of controls [164], [165], [168], [171], [174], [177], or at least within what may be considered a normal range of test performance. Therefore, these findings may be interpreted as reflecting neuroadaptation, perhaps indicating the recruitment of additional regions as a compensatory mechanism to maintain normal cognitive performance in response to chronic cannabis exposure [151], [152], [164], [166], [171], [172], [175], [179]–[181], [183], particularly within the prefrontal cortex area [151], [166], [168], [169], [171], [181], [183]. In this regard, the brain seems able to achieve some degree of reorganization, activating brain regions not usually needed to perform the cognitive task in response to an impaired ability of the normally engaged task network. Thus, it is feasible that drug-related compensatory mechanism may work for a period of time until it turns out to be insufficient and differences between groups become apparent. However, the impact of these subtle brain alterations on social, familiar and occupational life as well as its potential relationship with psychiatric disorders remains speculative.

A further important issue emerging out of this review is that few studies have investigated the effects of chronic cannabis use on the brain in adolescence subjects. In light of the popularity of cannabis among teenagers [1], [2] and recent data showing the potential neurotoxic effects of chronic cannabis use on the maturational brain [188], investigation of the possible long-term effects on brain structure and function in the adolescent population should be a priority both from the scientific and population health perspective [34], [188]. Future studies should consider the need for convergent methodology, replication of known facts with greater methodological rigor, and prospective large samples involving subjects of both genders across the life-span from adolescence to adulthood to delineate the evolution and reversibility of previously reported alterations.

### Limitations of the review

Results presented here have pointed out some important methodological differences that limit the generalisation of results and comparison between studies and have doubtless contributed to the slightly disparate array of findings. Despite the use of a strict definition of chronic cannabis user and robust application of inclusion and exclusion criteria in an attempt to avoid excessive heterogeneity between samples, studies often diverged on certain socio-demographic characteristics and cannabis use parameters, such as gender-bias, age of onset, lifetime use and abstinence period before the acquisition of imaging data. Moreover, it is well known that the THC content of smoked cannabis varies markedly between sources and preparations, with potency reported to have increased substantially over the past ten years [2]. Thus, comparability of earlier to later studies may not always be appropriate [44]. Furthermore, the exclusion of studies involving recreational and naïve cannabis users implies that the question of whether the brains of these subjects are adversely affected by cannabis is not addressed within the framework of the present review. Another important confounding factor is the inclusion of subjects with concurrent use of tobacco, which may affect neural activity as well as potentially interact with the effects of cannabis use [176]. In addition, it is known that co-morbid misuse of alcohol and other illicit drugs, such as cocaine and methamphetamine, may also be associated with significant neurobiological, neurocognitive and psychiatric abnormalities [189]. In the present review, although we excluded studies involving subjects with alcohol dependence, some included subjects with alcohol misuse (abuse [145], [179] or excessive consumption [150]), or reported differences in alcohol intake parameters despite alcohol consumption was within safe limits [143], [144], [147], [156], [157], [163], [164], [169], [170]. Moreover, given the relatively modest number of studies in the adolescent population, we included four studies which may involve some participants with co-morbid alcohol dependence [154], [155], [180], [181]. In all these studies, the interaction of alcohol with cannabis use, as well as its contribution to the brain effects cannot be ruled out. On the other hand, the exclusion of those with alcohol dependence, often highly co-morbid with cannabis use, may restrict the generalization of the results to the majority of chronic cannabis users [190].

With regard to other methodological limitations, some studies have reported modest sample sizes, sometimes below the threshold that would be currently regarded as acceptable (for instance, for PET or SPECT studies 10 subjects and for fMRI studies 15 subjects) [29]. In this regard, strategies for expanding data-sharing would be a welcome development in future research (i.e. The Function Biomedical Informatics Research Network [191] or the 1000 Functional Connectomes project [192], [193]). However, further obstacles must be addressed to make collaborative analysis efficient, such as between-site differences in scanners and data acquisition parameters, as well as pre- and post-processing schemes. The cross-sectional designs of most of the studies reviewed here complicated the interpretation of results as pre-existing morphological or functional alterations cannot be ruled out. Furthermore, studies that merely compare those subjects exposed to an environmental factor from those that are not, are likely to promote interpretation biases whereby study findings, irrespective of their direction, tend to be interpreted as detrimental. Longitudinal evaluations in larger samples may thus prove particularly useful. With regard to technical limitations, it is remarkable to note that the resting state studies did not control for spontaneous neutral activity and modulation of the BOLD signal, and the functional studies often reported different imaging methods and explored different brain functions using diverse cognitive paradigms, hampering the comparison between the studies. Hence, replication of previous results is critically important. Convergent methodology to sort out the current inconsistencies and controversies among studies would be important for future research in the field.

## Supporting Information

Table S1
